# Aesthetic Outcomes of Early Occlusal Loaded SLA Dental Implants with Hydroxyl Ion Modified Surface—A 12 Months Prospective Study

**DOI:** 10.3390/ma14216353

**Published:** 2021-10-24

**Authors:** Maciej Krawiec, Jakub Hadzik, Cyprian Olchowy, Marzena Dominiak, Paweł Kubasiewicz-Ross

**Affiliations:** Dental Surgery Department, Wroclaw Medical University, Krakowska St. 26, 54-207 Wrocław, Poland; maciej.krawiec@umed.wroc.pl (M.K.); cyprian.olchowy@umed.wroc.pl (C.O.); marzena.dominiak@umed.wroc.pl (M.D.); pawel.kubasiewicz-ross@umed.wroc.pl (P.K.-R.)

**Keywords:** dental implant, implant surface modification, early loading, marginal bone lost, periodontal index, pink esthetic score, white esthetic score, PES, WES

## Abstract

Background: Many efforts have been made recently to arrange a newer, more hydrophilic and more osteoconductive implant surface. One of the possible options in this matter is modification with hydroxyl ion. Materials and Methods: Forty implants with the diameters 3.5 and 4.0 mm were inserted as a single missing tooth restoration protocol in the frontal aspect of the maxilla. All implants were loaded early in a 4 week period. Prior to and during the surgery, the following indices were considered: height of keratinized tissue, the thickness of soft tissue, and the initial level of bone tissue. After 12 months, the implant and the tissues in its direct vicinity were evaluated once more with the following indices: marginal bone loss (MBL), height of keratinized tissue (HKT), probing pocket depth (PPD), pink and white aesthetics scores (PES, WES), as well as pain sensations combined with the procedure (VAS). All results were related to the diameter of the implant and thickness of periodontal biotype. Results: High aesthetic outcomes were reported regardless of soft tissue thickness and implant diameter. The VAS score was higher for the 4.0 implant group, and the thickness of soft tissue had no influence on VAS. In case of implantation in thin or soft tissue, higher MBL levels were reported (0.26 mm), while in case of a thick phenotype, MBL was 0.06 mm. Conclusions: Hydrophilic surface implants can be used for a protocol of early functional occlusal loading. The initial thickness of soft tissue does not influence aesthetic outcomes and does not raise pain perception, although it may elevate crestal bone resorption.

## 1. Introduction

Many factors affect the long-term success rate of implant treatment. One of the most crucial factors is the condition of peri-implant soft tissue. Sufficiently thick mucosa enables the creation of a barrier for bacterial ingrowth and supports a more natural implant emergence profile. Apart of easier to obtain proper hygiene maintenance around implant superstructure, the better esthetics outcome might be achieved in those cases [1].

Both the loading time and the type of prosthetic restoration play a role in supporting the soft tissue in implants’ direct vicinity. Previously used cemented restorations due to larger marginal micro-gaps, that could lead to more biofilm accumulation and a higher prevalence of peri-implant infections, were replaced with screw retained restorations [2]. Regarding loading time, previous prosthetic restoration of the implants in case of maxilla were provided within a period of 6 to 8 months. The reason was the need to establish osteointegration [3]. Osteointegration has been defined as a direct and functional connection between bone and an artificial implant. However, from a microscopic point of view, osteointegration manifests as filling the micro-gap between the bone bed and implant surface with bone tissue. As the bone tissue exhibits relatively low metabolism, it is a time consuming process [4,5].

However, immediate or early loading of a dental implant is especially desirable in implant treatment within the aesthetic zone. It would be beneficial if the healing period could be shortened without jeopardizing the success of the implant treatment [3].

Primary used smooth surface implants, disabled direct bone formation on their surfaces and prolonged osteointegration, secondary stability and furthermore the possibility loading. In the case of such surfaces, the bone overgrowth during implant healing was vectored in just one direction from the implant bed toward the implant surface. Introduction of a new type of rougher implant surface, at a micro scale, allowed for direct bone formation on the implant surface. This made the process of osteointegration bidirectional and shortened the time needed for it to occur [5,6]. While many believe that the possibility for further advances in implant surface microtopography has reached its limitation, significant effort nowadays is paid to the implant surface nanoscale [7,8,9].

Although newly prepared titanium surfaces are hydrophilic and are characterized by negative electrochemical potential and a stable thin layer of TiO_2_, they can be contaminated with an accumulation of non-polar hydrocarbons. This natural process first described by Att et al. was named titanium ageing, and results in changing the electrochemical potential and worsening the wettability of the implant surface [10]. For that reason, the chemical modification of the implant’s surface using hydroxyl ions was introduced as one of the options. With such modification, the titanium surface of an implant gains increased negative electrochemical potential.

### Aim of the Study

This study was designed to evaluate the hypotheses that implant diameter and soft tissue biotype influence (and if so in what proportion) the aesthetics outcomes, marginal bone loss, and pain sensation combined with the implant procedure. This was in cases of early loaded hydroxyl ion modified SLA (sand-blasted, large grit, acid-etched) implants in 12-month observation. The null hypothesis of the study was that thick, soft tissue biotypes and narrower implant diameters would not improve the value of periodontal indices, as well as aesthetic outcomes and marginal bone loss.

## 2. Materials and Methods

The present study was designed as a prospective study. The study was performed at Wroclaw Medical University Dental Clinical and Teaching facility. The study protocol was approved by a local ethics committee (registration number 229/2019). All patients gave two written consents: the first was general consent to have dental implants placed, and the second involved their participation in this study. The study has been conducted in full compliance with the Declaration of Helsinki, and personal data protection procedures (GDPR) were complied with.

### 2.1. Inclusion and Exclusion Criteria

This study is in addition to the authors’ previous study concerning the early loading of hydroxyl ion modified SLA implants [11], and here, we present the results of the soft tissue parameters that were evaluated.

Details on the specific inclusion and exclusion criteria and the exact clinical procedures were reported in the authors’ earlier study [11]. In brief, 40 adult patients needing single implant-supported crown rehabilitation, who could have immediate loading in the upper arch within the aesthetic zone, were enrolled into study. The patients could not have any active periodontal disease or API > 25%. Every participant was subjected to clinical and radiological examinations. The minimum residual bone crest needed to have a minimum width of 7 mm and a minimum height of 13 mm, so that the implant could be placed in the native bone. Furthermore, bone density in the region of the implant insertion should be D2 or D3 acc. to Misch et al. [12]. The randomization was performed on the day of surgery by drawing a ticket out of an envelope. The patients were randomly divided into 2 groups according to the implant diameters used (3.5 and 4.0 mm) and soft tissue thickness.

Group 1 (G2; *n* = 20 patients)—3.5 mm diameter implants were used
Group 2 (G3; *n* = 20 patients)—4.0 mm diameter implants were used

The guided bone or tissue regeneration procedures were not performed before nor during the implant placement. Furthermore, at least a 3-month healing period after extraction was established.

Remaining exclusion criteria were as follows:

1. systemic or local diseases that could compromise healing or osteointegration,
2. smoking,
3. bruxism,
4. pregnancy,
5. breastfeeding.

All exclusions were done through interview. Additionally, to exclude bruxism, intraoral examination was done to find any dental attrition, masticatory muscle overgrowth, or hyperactivity.

### 2.2. Protocol of an Experiment

The schedule of visits included the following:

1. Consultation visit: a precondition of the patient for the surgery, clinical and radiological examination CBCT (cone-beam computed tomography) (Galileos^®^ D3437, Sirona Dental, Erlangen, Germany), approximal plaque index (API), height of keratinized tissue (HKT) assessment;
2. Implantation: intraoperative and postoperative RVG (radiovisiography—Planmeca OY, Helsinki, Finland), gingival biotype assessment thick/thin;
3. Four weeks after the implantation: intraoral scan, screw-retained prosthetic, RVG;
4. Twelve months after the surgery: clinical evaluation (HKT, probing pocket depth (PPD), Visual Analogue Scale (VAS), pink esthetic score (PES) and white esthetic score (WES)) and radiological assessment (RVG and CBCT).

### 2.3. Implants

The cylindrical dental implants Thommen Innicell^®^SPI Element MC Innicel (Thommen Medical AG, Grenchen, Switzerland) were used for the surgery. The superhydrophilic surface of the implants was acquired through NaOH conditioning using the Apliquiq system (Thommen Medical AG, Grenchen, Switzerland). The length of the inserted implants ranged from 8 mm to 11 mm and was conditional on the height of the bone base, while the diameter of the implant was determined by the width of the alveolar processes.

### 2.4. Surgical Phase

**Figure 1:**
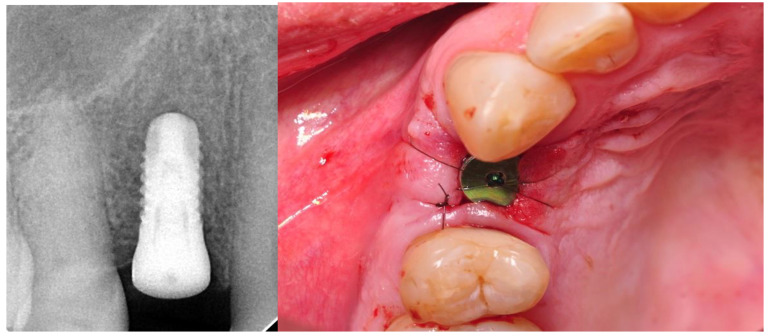
Implant with the healing abutment placed in bicuspid region and left for open healing.

The implant surgery included the antibiotic cover of one-shot therapy—1 dose of clindamycin 600 mg (MIP Pharma, Gdansk, Poland). Choice of implant was based on radiological examination performed during the preconditional visit. The implant’s surface was conditioned due to insertion with an Apliquiq applicator just before the surgical procedure. Local infiltration anesthesia was provided using Septanest 1:100,000 (SEPTODONT 58, Saint Maur des Fossés, France) with the application of the Wand STA device (Milestone Scientific, Inc. Roseland, NJ, USA). First, a diamond drill on the high-speed hand piece was used for deepithelialization. Then, H-shaped papilla-preservation incision, shifted palatially with a blade no. 15C was done. The implant bed was performed following manufacturer recommendations. During that stage, implants were lifted from the Apliquiq applicator. Subsequently, each implant was inserted at bone level according to the procedure provided by the manufacturer. The partially deepitalized flap was repositioned and stabilized with 5-0 simple interrupted sutures (Seralene^®^, Serag Wiessner, Naila, Germany). At the end of the surgery, an RVG image was done to control the correctness of the implant insertion (Figure 1). The X-ray tubehead was aimed at right angles (vertically and horizontally) to both the implant and the sensor. A paralleling device was used for this purpose. The surgeries were performed by M.K and J.H. Postoperative recommendations included analgesic and anti-inflammatory treatment with Nimesil (Laboratories Menarini SA Barcelona, Spain), 200 mg/per day, and rinsing the oral cavity with Eludril Classic (Pierre Fabre S.A Paris, France) 3 times a day.

### 2.5. Prosthetic Phase

**Figure 2:**
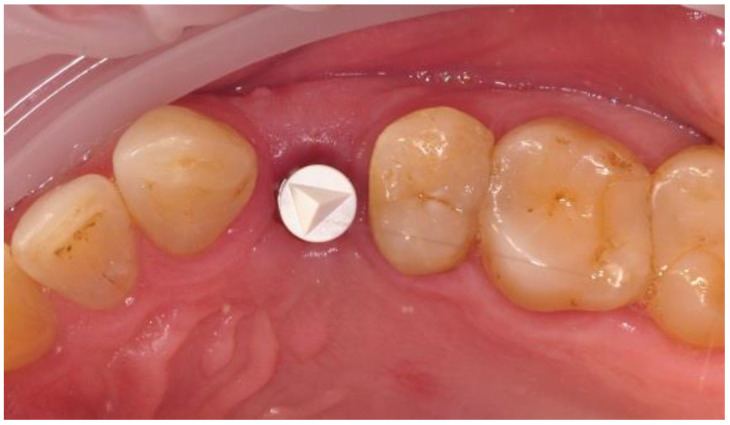
Implant with scanbody prepared for intraoral scan.

**Figure 3:**
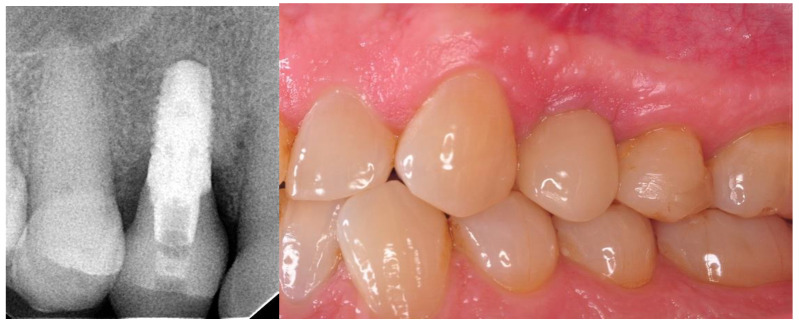
One-year follow-up. Periapical RTG and clinical photography. Implant loaded with the screw-retained crown.

The prosthetic restoration stage was conducted 4 weeks after the implant placement surgery and was done by M.K, and J.H Patients with no signs of inflammation in direct vicinity to the implant were allowed to participate in the prosthetic protocol. Screw-retained implant crowns made of lithium disilicate glass-ceramics, IPS e max CAD LT, were used as prosthetic restorations. After the removal of the healing abutment, the implant bed was cleaned. The scans were taken with an intraoral scanner Sirona Cerec AC Bluecam (DentsplySirona, York, PA, USA) (Figure 2). Subsequently, the surface was etched using IPS Keramik etching gel (Ivoclar Vivadent AG, Schaan, Liechtenstein) and sandblasted using Ti base (DentsplySirona, York, PA, USA), and then the crown was fixed using Monobond Plus bonding agent (Ivoclar Vivadent AG, Schaan, Liechtenstein) and Multilink Hybrid Abutment cement (Ivoclar Vivadent AG, Schaan, Liechtenstein). The crown was then screwed onto the implant with a torque of 25 Ncm. The occluding relations were controlled using articulating paper (Bausch^®^, Cologne, Germany) with a thickness of 200, 80, and 8 μm. Access to the retaining screw was closed with Gradia composite (GC Corporation, Tokyo, Japan), and an RVG image was taken (Figure 3). The patients were instructed on proper hygiene around the dental implant.

### 2.6. MBL (Marginal Bone Loss) Assessment Using the Radiological Examination

Prior to the surgery and during 12 months of follow-up, CBCT was performed to assess the marginal bone loss (MBL). The MBL was calculated as follows: first, dimensions were calibrated by the known parameters of implant diameter and length. Starting from the implant shoulder, distances were measured to the mesial and distal points of bone to implant contact, parallel to the implant axis. All measurements were taken by C.O., a member of the research group who was not involved directly in the preparation of the implant.

### 2.7. Periodontal Parameters Measurements

The following clinical parameters were assessed during the study:

-PPD at 4 points (mesial, distal buccal and palatal), measured to the nearest 1 mm using a periodontal probe (Williams Color-Coded Probe, Hu-Friedy Mfg. Co., Chicago, IL, USA);

-HKT using a periodontal probe;

-The thickness of soft tissue was assessed using gingival transparency method by visibility of the underlying periodontal probe, regarding ≥ 2 mm as a thick gingiva.

### 2.8. Aesthetic Evaluation

The measurements of PES (Pink Esthetic Score) and WES (White Esthetic Score) were done at 12 months according to Belser et al. [13]. Briefly, standardized digital photographs (CanonEOS 650 with ring flash, Canon Inc., Tokyo, Japan) of the aesthetic zone were taken. The peri-implant soft tissue was graded based on five categories: mesial papilla, distal papilla, curvatures of the facial mucosa, level of the facial mucosa, and root convexity/soft tissue color and texture. To evaluate WES of the visible portion of the implant restoration, five categories were graded: tooth form, outline/volume of clinical crown, color, surface texture, and translucency/characterization. Each of the 5 topics of PES and WES was graded with a 0-1-2 score and resulted in lowest 0 and highest 10 for each of two scores consequently.

### 2.9. VAS (Visual Analogue Score) Assessment

After a 1-week follow-up, each participant was asked to evaluate his or her pain sensation after the procedure, using a VAS ruler, with zero representing no pain and 10 the worst pain the patient had ever experienced.

### 2.10. Statistical Analyses

To answer the research questions, statistical analyses were performed using IBM SPSS Statistics 25 software (IBM, New York, PA, USA). The software was used to analyze the basic descriptive statistics together with the U Manna–Whitney test. First, descriptive analysis was done using the *χ*^2^ test. The statistical differences were tested between the 3.5 and 4.0 diameters, and between thick and thin biotype groups. Because group 3.5 and 4.0 were equal, the parametric tests could be used. Hence, the thick and thin biotype groups were not equal, the Pearson’s *χ*^2^ test was used.

The relationships between continuous variables were examined by calculating Pearson’s linear correlation coefficient. The value of α = 0.05 was assumed as the significance level.

To check the distribution of continuous variables and to study their compliance with a normal distribution, basic descriptive statistics were used and the Shapiro–Wilk test of normal distribution was performed. For nominal variables, the frequency and the percentage of individual values in the entire observation pool were calculated.

## 3. Results

### 3.1. General Data

Out of 40 implants placed in the surgical phase, each implant achieved osteointegration and consequently high level of secondary stability and was admitted in the prosthetic phase. Furthermore, all implants successfully survived for the 12-month follow-up period.

### 3.2. Results of MBL

We achieved relatively low levels of MBL for all implants 0.14 mm (±0.24). However, it was lower within the thick gingival biotype group (0.06) when compared to the thin biotype (0.26). The differences between these two groups were not statistically important. There were also no statistically important differences in that parameter between 3.5 and 4.0 group (0.26 mm (±0.31) vs. 0.14 mm (±0.24)) (Table 1 and Table 2).

### 3.3. Results of Periodontal Parameters Measurements

**Figure 4:**
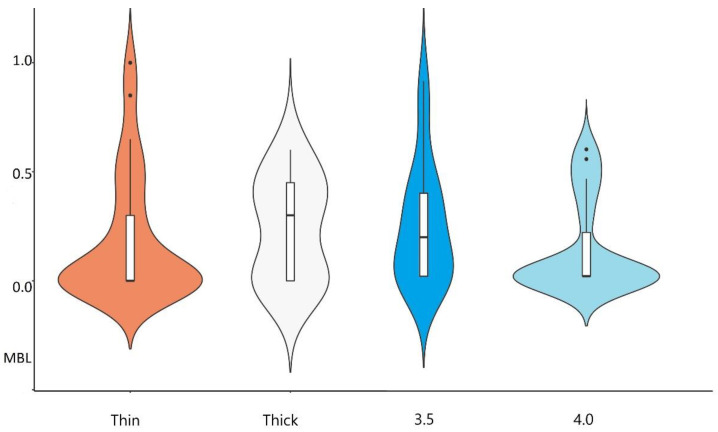
The graphical presentation as a boxplot of MBL results among groups of thick/thin biotype and 3.5/4.0 diameter implants.

The average PPD in 3.5 and 4.0 group implants were comparable: 2.17 mm (±0.53) vs. 2.04 mm (±0.37), with no statistically important differences between them. The thickness of soft tissue had no influence on PPD levels as the results of PPD for thick and thin biotype group were the same (2.1 mm) (Table 2). The results of HKT measurements showed statistically important differences between thin and thick biotype group. The HKT slightly improved after 12 months in the thin biotype group, from 3.64 mm (±0.91) to 3.66 mm (±1.01), while the thick biotype group remained stable at 4.64 mm (±1.02) (Table 2). The diameter of the implant had no influence on HKT measurements as there were no statistically important differences between 3.5 and 4.0 groups (Table 1, Figure 4).

### 3.4. Results of Aesthetic Evaluation

**Figure 5:**
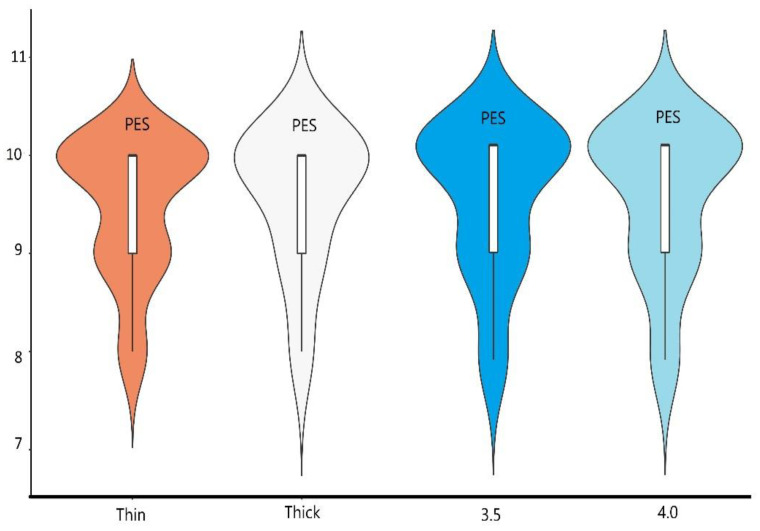
The graphical presentation as a boxplot of PES results among groups of thick/thin biotype and 3.5/4.0 diameter implants.

**Figure 6:**
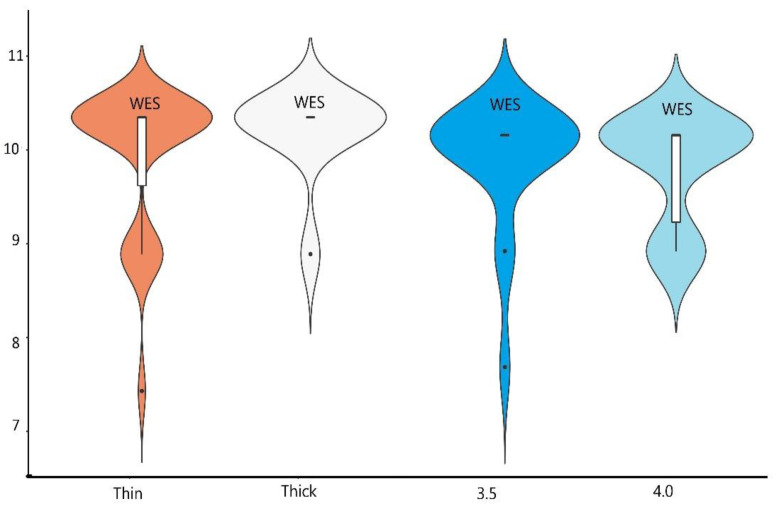
The graphical presentation as a boxplot of WES results among groups of thick/thin biotype and 3.5/4.0 diameter implants.

Good aesthetic outcomes were achieved for all implants (Table 1, Figure 1). While there were no differences in pink aesthetics (9.5) for both 3.5 and 4.0 groups, slightly better results were achieved for 3.5 implants in white aesthetics 9.78 vs. 9.72. Thicker biotype of soft tissues moderately improved pink and white aesthetics (9.48 vs. 9.56 and 9.7 vs. 9.89), but with no statistically important differences (Table 2, Figure 5 and Figure 6).

### 3.5. Results of VAS Score

There were no statistically important differences in pain sensation after the procedure for both 3.5 and 4.0 groups. The thicker biotype of soft tissues had a slightly smaller VAS score, however without a statistically important difference: 1.27 vs. 1.79.

## 4. Discussion

The first clinical trial that proposed immediate or early implant loading was conducted in 1990 [14]. Since then, many others studies have proven the safety of such modified treatment protocols, and, moreover, the paradigm of osteointegration necessity for implant loading has been challenged [15,16].

Previously, the most commonly used parameters for evaluating success rate of the implant treatment were related to the implant, the bone and soft tissue in the direct vicinity of the implant, and the prosthesis, apart from the subjective assessment of the patient [17]. These parameters are related to the tissue stability, which influences the progression of marginal bone loss. For decades, the concept of marginal bone loss implied the possibility of 2 mm crestal bone loss in the first year, and up to 0.2 mm every year around the implant neck after functional loading.

However, the recently introduced zero bone loss concept seems to change the paradigm of bone resorption consideration and features a newer point of view on that matter. It is believed that, due to sufficiently thick mucosa, the bone loss around the implant neck can be prevented [18]. The last study of this scope proved the threshold point for soft tissue thickness at 2.88 mm, as such thick soft tissue can prevent bone loss [19,20]. In the present study we decided to exclude patients with previous augmentation procedures, including soft tissue grafts. As a result, in only 11 cases the thick biotype of the gingiva was spotted, while in the majority of cases (29,) we reported a thin biotype. However, even so, the relative low levels of MBL for both thin and thick biotype was reported. None of the patients, regardless of the thickness of soft tissue biotype, even approached the abovementioned proposed levels of MBL. The reason for that finding—in our consideration—might be a relative short observation period. Another explanation for the relatively low MBL could be the fact that most of the patients were characterized by high levels of HKT. This means that even if the soft tissue around the implant’s neck was thin, it was at least attached and highly keratinized. Finally, early loading of the implant allows for establishing a peri-implant junction sooner, contributing a biological seal and preventing peri-implant sulcus from bacterial ingrowth. This can provide a higher chance to maintain crestal bone level [21,22,23]. However, what is worth noticing is that the group of thick biotype was still characterized by statistically important lower MBL levels in comparison to thin biotype.

One of the biggest challenges in implant restoration within the frontal aspect of the maxilla is the aesthetic outcome. However, the primary focus of early literature on maxillary anterior implant outcomes was based on survival parameters, with a lack of information regarding aesthetically relevant parameters. Previously used subjective evaluation was carried out using patient perceptions of the aesthetic outcome, measured by specific questionnaires in which patients express their degree of satisfaction or dissatisfaction. This was replaced with several more reliable objective indices introduced to assess clinician-mediated aesthetic outcomes for single-tooth implant restorations in the aesthetic zone [24,25,26]. One of the most objective tests to assess aesthetics of the prosthetic restoration is the pink (PES) and white aesthetics scores (WES). PES was first proposed by Furhauser and was lately redefined by Belser et al. [13,27].

The PES according to Belser et al. includes the mesial papilla, distal papilla, soft tissue level, curvature of the facial mucosa, and root convexity/soft tissue color and texture [13]. The height of interdental papilla is especially considered a crucial factor in aesthetics, as it is the most commonly affected part of peri-implant soft tissue. Scientific data demonstrated that the appearance of the papilla in the interproximal area of an implant site is largely dependent on the vertical distance between the alveolar bone crest and contact point, as well as the horizontal distance between the implant and the neighboring implant/tooth [28,29]. A score of ≥6 (out of a maximum of 10) for either PES or WES and ≥12 (out of a maximum of 20) for PES/WES combined are generally considered satisfactory.

In the present study, generally good aesthetic outcomes were achieved as the overall PES and WES scores were 9.5 and 9.75 and almost reached their maximum. It also shows high average levels of interproximal papilla height. It was suggested that soft tissue thickness might influence the aesthetic scores. However, we did not report such findings. In our study, high level of PES and WES was achieved even if in the vast majority of the cases the soft tissue profile was reported as thin. There can be some explanations for this phenomenon. First, in our consideration, it can be a result of average to good crestal bone level preservation and the supporting function of bone tissue on interproximal papilla height. Second, it is suggested that the fixed prosthesis provided an early contribution to physical support for soft tissue in direct contact around the implant and may result in better soft tissue condition.

Lastly, we decided to include VAS score as a final parameter to be considered in the following study. The reason for that is, apart from good aesthetics outcomes and the general success rate of the implant therapy, any harmful sensation combined with the procedure or healing period might influence final patient satisfaction. Pain associated with the procedure is also one of the major reasons for neglecting or postponing further dental therapy. Generally, pain combined with an early loading protocol as proposed in our study was considered by the patients as acceptably low. The wider implant procedure generated higher pain sensations as the VAS score amounted to 1.75 (±1.33) for the 4.0 group and 1.53 (±0.96) for the 3.5 group. The explanation for that finding might by either the need to apply one more drill in the wider implant protocol as well as more time-consuming procedure in that case. The thickness of soft tissue seems not to influence the healing period in the same proportions as in both thick and thin mucosa similar VAS results were achieved.

The literature is missing reports engaging the diameter of the implant or the thickness of the soft tissue with pain related sensations. Generally, as found in the literature, the pain sensations combined with implant insertion might be much higher than reported in the present study. Kim et al. found average VAS score as measured during the procedure to be 4.33 (±3.30) [30], while Youk et al. reported VAS score at 3.34 for non-template guided implant procedures [31].

### Limitations of the Study

The main limitation of the present research is the lack of control group of conventionally loaded implants and early loaded implants with no hydroxyl ion modification. The reason for not including this control group is that early loading protocol with available, commercially non-modified in nano-scale surface implants has been evaluated before by other reports. Other limitations are the short follow-up period. Further research should be conducted to control the achieved results and to evaluate the osteointegration process at its most critical period.

## Figures and Tables

**Table 1:** Descriptive statistics and the normality test to determine distribution for selected variables for group 3.5 and 4.0. M-Mean, SD-Standard Deviation, t-t distribution, p-probability value.

Variable	Group3,5		Group 4,0		t	p	d Cohena
	M	SD	M	SD			
MBL	0.26	0.31	0.14	0.24	1.30	0.203	0.43
HKT 0	4.11	1.15	3.75	1.16	0.96	0.344	0.31
HKT 1	4.00	1.30	3.85	0.88	0.43	0.671	0.14
PPD	2.17	0.53	2.04	0.37	0.82	0.417	0.27
VAS	1.53	0.96	1.75	1.33	−0.60	0.554	−0.19
PES	9.50	0.71	9.50	0.71	0.00	1.000	0.00
WES	9.78	0.55	9.72	0.46	0.33	0.744	0.12

**Table 2:** Descriptive statistics and the normality test to determine distribution for selected variables for group with thin and thick biotype. Differences important statistically are highlighted in red. Me-median, IQR-interquartile range, M-Mean, SD-Standard Deviation, p-probability value.

Variable	Biotype					
	Thin (*n* = 27)					
	Mean rank	Me	IQR	M	SD	p
MBL	17.76	0.00	0.35	0.19	0.29	0.472
PPD	18.13	2.00	0.75	2.11	0.49	0.709
PES	18.20	10.00	1.00	9.48	0.7	0.736
WES	17.81	10.00	1.00	9.7	0.54	0.35
HKT0	17.57	4.00	1.00	3.64	0.91	0.028
HKT1	17.79	4.00	1.00	3.66	1.01	0.014
VAS	1.79	1	1	1.79	1.29	0.464
	Thick (*n* = 13)					
	Mean rank	Me	IQR	M	SD	p
MBL	20.72	0.30	0.45	0.24	0.24	0.472
PPD	19.61	2.25	0.56	2.1	0.36	0.709
PES	19.39	10.00	1.00	9.56	0.53	0.736
WES	20.56	10.00	0.00	9.89	0.33	0.35
HKT0	26.18	5.00	2.00	4.64	1.43	0.028
HKT1	27.64	5.00	1.00	4.64	1.02	0.014
VAS	1.29	1	1	1.27	0.47	0.464

## Data Availability

The data presented in this study are available on request from authors.
